# Use of Ionic Liquids in the Enzymatic Synthesis of Structured Docosahexaenoic Acid Lyso-Phospholipids

**DOI:** 10.3390/molecules30030728

**Published:** 2025-02-06

**Authors:** Ernestina Garcia-Quinto, Jose M. Guisan, Gloria Fernandez-Lorente

**Affiliations:** 1 Laboratory of Microbiology and Food Biocatalysis, Institute of Food Science Research (CIAL, CSIC-UAM), Nicolás Cabrera, 9, UAM Campus, Cantoblanco, 28049 Madrid, Spain; ernestina.garcia@csic.es; 2 Department of Biocatalysis, Institute of Catalysis and Petrochemistry (ICP, CSIC), Marie Curie, 2, UAM Campus, Cantoblanco, 28049 Madrid, Spain; jmguisan@icp.csic.es

**Keywords:** DHA lysophosphatidylcholine (DHA-LPC), immobilized lipases, ionic liquids, esterification, 1-methyl-3-octylimidazolium tetrafluoroborate (MOIM-BF_4_)

## Abstract

Recent studies have shown that DHA supplementation in the form of phospholipids effectively increases DHA levels in the brain, including DHA lysophospholipids. This research explores a method to produce DHA lysophosphatidylcholine (DHA-LPC) using lipases and phospholipases immobilized on Immobeads-C18 with maximal enzyme loading. The esterification of glycerophosphatidylcholine (GPC) and DHA was studied with ionic liquids as alternatives to traditional solvents, with 1-methyl-3-octylimidazolium tetrafluoroborate (MOIM-BF_4_) providing the highest yield due to its ability to increase the solubility of GPC. The reaction parameters were modified to establish a molar ratio of GPC to DHA of 1/10. A maximum DHA-LPC yield of 80% was achieved in 48 h, with a formation rate of 20.06 (mg/mL.h) × g. The Quara^®^ LowP biocatalyst (QlowP-C18) maintained 100% activity during the first three cycles and produced 788 mg of DHA lysophospholipid. The use of 50% MOIM-BF_4_ improved the stability of the biocatalyst, and NMR confirmed that the product was the sn1-DHA-LPC isomer.

## 1. Introduction

Long-chain ω-3 PUFAs, especially DHA, play a fundamental role in the development and maintenance of the nervous system. In recent years, their consumption has been shown to be associated with a lower risk of developing neurodegenerative diseases [1,2]. Several studies have observed that DHA, when supplied in the form of phospholipids, has a significantly higher bioavailability than in the form of triglycerides [3]. This is because phospholipids (PL) follow a simpler digestion and distribution process than triglycerides (TAG) [4]. Due to their amphipathic nature, PLs have higher solubility in aqueous media and can diffuse through the lipid bilayers of cells. In particular, several authors have pointed out that the lysophosphatidylcholine molecule is the preferred transporter of DHA to cross the blood–brain barrier and deliver the fatty acid to the brain [5,6,7].

Lipases and phospholipases have generated great interest in the food industry for their applications as biocatalysts in the modification and production of lipids structured with ω-3 polyunsaturated fatty acids However, there are few examples in the literature on the synthesis of phospholipids using immobilized lipases/phospholipases. Mainly, the synthesis of lysophosphatidylcholine (LPC) from glycerophosphatidylcholine (GPC) is described, either by direct esterification with free fatty acids [8,9] or by transesterification with fatty acid vinyl esters [10,11,12]. In a recent study, DHA PLs were synthesized through transesterification mediated by a lipase soluble in aqueous phases. Lipase B from *Candida antarctica* (CALB) stood out as the best biocatalyst, achieving a DHA incorporation of 34.8% and a PL yield of 80% [13]. One of the problems when modifying GPC in anhydrous solvent-free systems is its low solubility in the reaction medium since it is a highly polar molecule, easily soluble in water and polar solvents, but almost insoluble in completely anhydrous organic media. Moreover, the instability of enzymes immobilized in anhydrous hydrophobic media (e.g., oils) [14] may be an additional drawback for the development of enzymatic processes in solvent-free systems. Therefore, highly active and stable biocatalysts that can withstand various reaction conditions are necessary to achieve high synthetic yields in the synthesis of structured DHA phospholipids [15]. Adsorption of lipases on highly hydrophobic supports involves the open form of lipases, allowing for the immobilization, purification, hyperactivation [16], and stabilization of lipases in a single step [17]. Abreu Silveira et al. increased the activity of TLL (*Thermomyces lanuginosus lipase*) 17-fold through interfacial adsorption on various hydrophobic supports [14]. Cejudo-Sánchez et al. stabilized RML (*Rhizomucor miehei* lipase) by more than 100-fold compared to the soluble form of the enzyme [18]. In a recent investigation, García-Quinto et al. demonstrated that NS40 and PALA lipases, immobilized by hydrophobic adsorption on the Immobeads-C18 support, achieved 100% DHA release in only 24 h, due to their high enzyme loading [19].

The incorporation of organic solvents into anhydrous reaction systems is often presented as an effective option to solubilize substrates with different polarities, achieving adequate homogenization of the medium [20]. In a study on the enzymatic synthesis of lysophosphatidylcholine, it was observed that the addition of small amounts of the solvent dimethylformamide, which mimics water, significantly improved the reaction rate and product yield [8]. However, many common organic solvents can denature proteins, distorting the structure of lipases and affecting their activity and stability [21].

As an alternative to conventional chemical solvents, ionic liquids (ILs) have been developed as a new class of environmentally friendly solvents. ILs, also known as liquid salts, are substances that remain liquid at relatively low temperatures and are composed of positive and negative ions [22]. ILs are recognized as “green solvents” due to their low volatility and properties such as chemical inertness, thermal stability, ease of degradation, non-toxicity, non-flammability, reusability, and ease of separation and purification of reaction products. In addition, they have a wide boiling point range [23]. These properties reduce waste generation, making the processes more economical and environmentally friendly [24,25].

According to the literature, enzymes in the presence of ILs can show enhanced activity, stability, and selectivity compared to conventional organic solvents [26,27]. Nascimento et al. studied how lipase from *Aspergillus niger* showed enhanced activity in ILs with a short cationic alkyl side chain, namely 1-butylimidazolium chloride ([bmim]Cl) and 1-hexyl-3-methylimidazolium chloride ([hmim]Cl) [28]. In another study, the lipase-catalyzed transesterification reaction was analyzed, showing that both the reaction rate and enantioselectivity were dependent on the IL anion. The best results were obtained with 1-butyl-3-methylimidazolium tetrafluoroborate ([C4mim][BF4]) and hexafluorophosphate salt ([C4mim][PF6]) [29].

**Figure 1:**
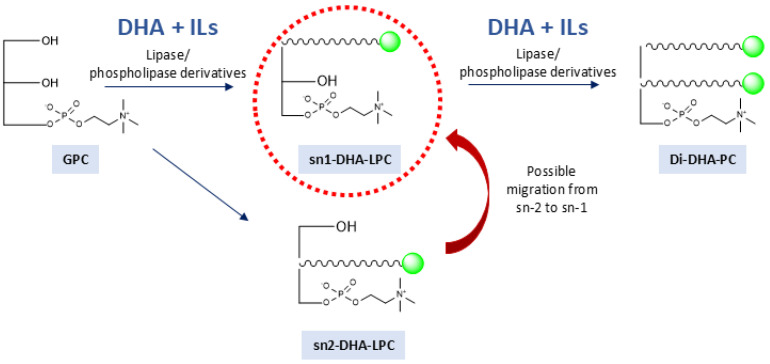
Schematic representation of the enzymatic synthesis reaction of DHA-structured phospholipids catalyzed by immobilized and regioselective lipases and/or phospholipases in anhydrous media containing ionic liquids.

As illustrated in Figure 1, this study investigates the addition of various ILs to the esterification reaction of GPC and DHA catalyzed by immobilized lipases. Unlike previous investigations, which have not explored the systematic use of ILs in this type of reaction, this work aims to evaluate their potential to improve the performance of the process. The main innovation lies in the use of ILs to improve GPC solubility, stabilize the biocatalyst, and promote a more sustainable process. It also aims to optimize the amount of GPC and IL to minimize the excess of DHA in the medium, reducing waste and improving profitability.

## 2. Results and Discussion

### 2.1. Immobilization of Lipases/Phospholipases on Hydrophobic Support with Maximum Enzyme Loading

The five lipases and two phospholipases were immobilized on the Immobeads-C18 support by hydrophobic interfacial adsorption, aiming for the highest possible enzyme loading. The enzyme loading per gram of support varied depending on the enzyme and the immobilization yield achieved. TLL, PALA, and NS40 achieved 180 mg of protein per gram of support, with an immobilization yield of 90% after 72 h. CALB, NOVO, and phospholipase LECI showed a final enzyme loading of 170 mg per gram of support after 48 h of continuous agitation. Finally, phospholipase QlowP reached a loading of 160 mg per gram of support after 72 h.

### 2.2. Use of Ionic Liquids as Reaction Media for the Synthesis of DHA Phospholipids

In this study, we investigated the incorporation of 30% ionic liquid into the reaction medium for the synthesis of DHA phospholipids, with the aim of addressing the limitations encountered in solvent-free reactions previously explored by our research group [30]. Previous research has established that, under solvent-free conditions, the predominant product formed is the di-substituted phospholipid. However, our results demonstrate that the addition of 30% MOIM-BF_4_ significantly alters the course of the esterification reaction. For this purpose, MOIM-BF_4_ ionic liquid was initially selected, and the esterification reaction was carried out using five different lipases and two phospholipases, all immobilized on the Immobeads-C18 support with maximum enzyme loading.

**Figure 2:**
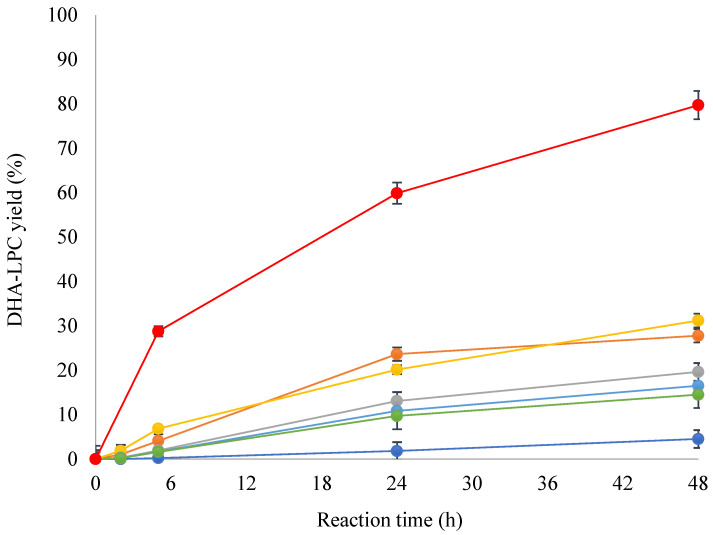
Reaction yield for DHA-LPC catalyzed by several lipases and phospholipases immobilized on Immobeads-C18 in 30% MOIM-BF_4_ medium: TLL (dark blue), CALB (orange), NS40 (gray), PALA (yellow), NOVO (light blue), LECI (green), and QlowP (red). Reaction conditions: amount of derivative: 10% w/w of total substrate weight, molar ratio of substrate: 1 mole of GPC to 14 moles of DHA (1/14), temperature: 60 °C, and stirring speed: 150 rpm.

As illustrated in Figure 2, the presence of the ionic liquid facilitates the synthesis of the mono-substituted phospholipid, DHA-LPC, but does not promote its conversion to the di-substituted phospholipid. This effect is consistently observed in all the enzymes studied, suggesting that the incorporation of the ionic liquid into the reaction medium induces conformational changes in the enzymes. Among the biocatalysts evaluated, the phospholipase QlowP-C18 showed superior performance, achieving 80% conversion to DHA-LPC within 48 h, whereas the other enzymes demonstrated significantly lower conversions; at best, PALA-C18 only achieved 31% conversion in the same time period.

Considering these results, QlowP-C18 is confirmed to be the most promising biocatalyst for the synthesis of monosubstituted DHA phospholipids due to its characteristics as a phospholipase [31], its natural substrate being a phospholipid. Unlike LECI phospholipase, QlowP is a novel thermostable and acid-stable phospholipase from *Talaromyces leycettanus*, produced in *Aspergillus niger*. Moreover, the thermal denaturation temperature of QlowP is 17 °C higher than that of LECI phospholipase, making it active at 70 °C [32]. Therefore, these characteristics suggest that QlowP-C18 could be the most suitable and active enzyme to efficiently catalyze the synthesis of monosubstituted DHA phospholipids under conditions of elevated temperature (60 °C), acidic environments (DHA) and with ionic liquid (MOIM-BF_4_).

### 2.3. Effect of Ionic Liquids on the Synthesis of Structured Phospholipids

**Figure 3:**
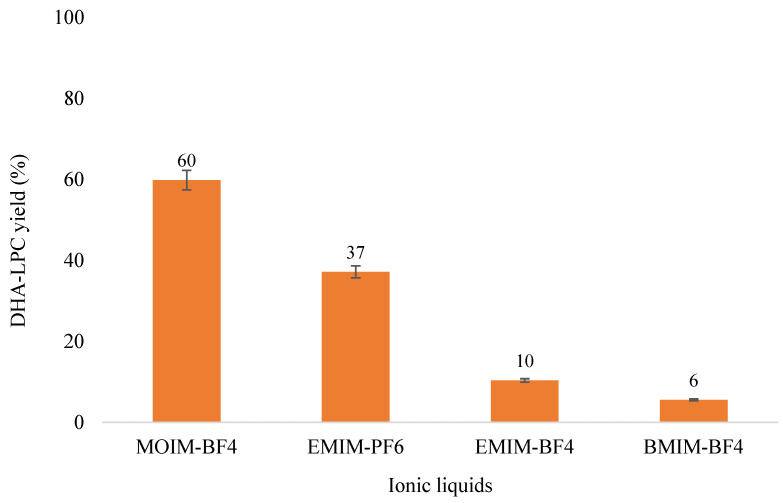
Effect of the addition of 30% of various ionic liquids on the 24-h reaction yield for DHA-LPC catalyzed by QlowP-C18, with a substrate molar ratio of 1/14 (GPC/DHA) and at 60 °C.

The addition of 30% of various ionic liquids to the esterification reaction of GPC and DHA catalyzed by QlowP-C18, which is the most active enzyme derivative previously validated, was investigated. Four ionic liquids (EMIM-Cl, EMIM-Otf, EMIM-Ac, and EMIM-AlCl_4_) did not allow the enzyme to catalyze the reaction efficiently due to their high viscosity or low solubility in the reaction medium, leading to their exclusion from the study. In contrast, the remaining four ionic liquids effectively facilitated the catalysis of the reaction, as shown in Figure 3. According to the results, MOIM-BF_4_ is the most suitable ionic liquid for the esterification reaction, as it provides the highest yield of DHA-LPC, reaching 60% at 24 h as the only product of the reaction.

On the other hand, the polarity of the reaction medium is crucial as it affects enzyme activity and substrate solubility. According to the literature, highly polar ionic liquids could affect enzyme stability by removing the essential water layer [33,34] or interacting with it to inactivate the enzyme [35]. For example, ionic liquids with BF_4_^−^, which are highly hydrophilic, may have this effect. However, it is important to note that these results have been observed predominantly in studies with enzymes in solution. In our study, we used immobilized enzymes, which prevent denaturation and thus ensure greater stability under the experimental conditions. In contrast, ionic liquids containing PF_6_^−^, which have hydrophobic characteristics, stabilize lipases by protecting their hydration layer [27] or by inducing a beneficial conformational change [26].

Furthermore, based on the results shown in Figure 3, it appears that shorter alkyl substituents in the solvent, such as a methyl group, correlate with lower viscosity, as they create a less compact structure and facilitate molecular mobility, thus improving reaction performance. In this regard, ionic liquids with a methyl substituent are preferred over those with an ethyl substituent, which, in turn, are favored over those with a butyl substituent. In this context, the methyl group of the positively charged ion, combined with the hydrophilic nature provided by the counterion BF_4_^−^, could enhance the solubility of GPC, consequently increasing the formation of the DHA-LPC product.

### 2.4. Effect of the Percentage of MOIM-BF_4_ on DHA Lysophospholipid Synthesis

Given the positive effect observed with the addition of MOIM-BF_4_ in the previous section, different percentages of this ionic liquid were investigated. The objective was to maximize the solubility of GPC and reduce the amount of DHA used in the reaction medium, making the process more economically viable. Table 1 presents the initial reaction rates obtained for the formation of DHA lysophospholipid as a function of the percentage of MOIM-BF_4_ used. It also shows the molar ratio between the substrates (GPC/DHA) in each case, indicating that with a higher percentage of ionic liquid, less DHA is used relative to GPC.

According to the results, as the percentage of MOIM-BF_4_ in the medium relative to DHA increases, the rate of DHA-LPC formation increases up to a 50%:50% ratio. Beyond this ratio, the rate begins to decrease. This suggests that the use of 50% MOIM-BF_4_ provides the highest reaction yield, possibly due to increased solubility of GPC in the medium or improved enzyme stability. In addition, the results indicate that the reaction requires an excess of DHA in the medium, approximately ten times more than theoretically necessary (1 GPC/1 DHA), as observed in the ratio of 50% MOIM-BF_4_ and 50% DHA. This excess facilitates interactions between the substrates, allowing the catalyst to efficiently carry out the esterification of at least one position of the GPC backbone, which is better dissolved due to the presence of the ionic liquid in the reaction medium.

### 2.5. Effect of the Molar Ratio of GPC/DHA Substrates with 50% MOIM-BF_4_

After reaching the maximum yield of the monosubstituted DHA phospholipid DHA using 50% MOIM-BF_4_, the molar ratio of GPC to DHA was further investigated. The objective was to improve the yield of the monosubstituted phospholipid by increasing the concentration of GPC. Various amounts of GPC, including 30 mg, 90 mg, 180 mg, and 300 mg, were tested, maintaining a constant total reaction volume of 2.5 mL (1.25 mL DHA and 1.25 mL MOIM-BF_4_) in all experiments. Accordingly, the molar ratios of GPC to DHA were set as 1/31, 1/10, 1/5, and 1/3, respectively. This indicates that for each mole of GPC, there are 31, 10, 10, 5, and 3 moles of DHA in the different experimental setups.

**Figure 4:**
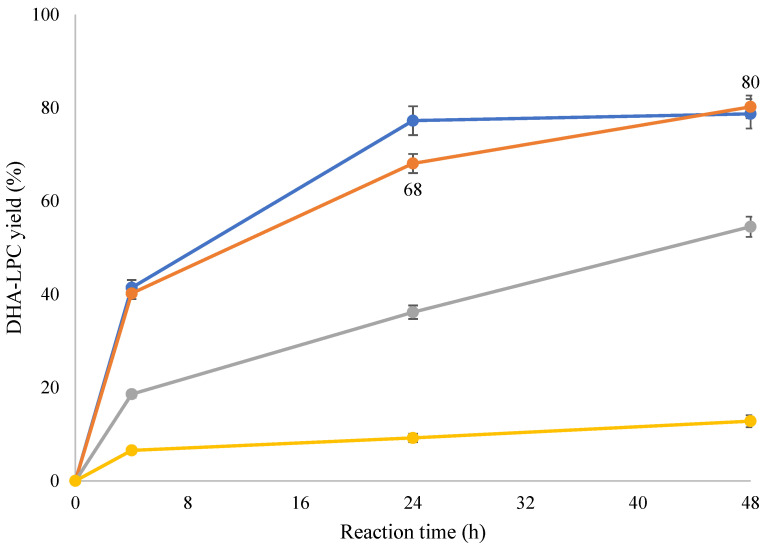
Course of the esterification reaction of DHA and GPC catalyzed by QlowP-C18, in a medium containing 50% MOIM-BF_4_ at 60 °C. GPC/DHA molar ratio: 1/31 is 30 mg GPC (blue), 1/10 is 90 mg GPC (orange), 1/5 is 180 mg GPC (gray), and 1/3 is 300 mg GPC (yellow).

Figure 4 shows the course of the esterification reaction catalyzed by QlowP-C18 with the different amounts of GPC under study. It is observed that, in general, as the amount of GPC increases, the production of DHA-LPC decreases due to the lower solubilization of GPC at higher concentrations. However, with the use of 50% MOIM-BF_4_, a maximum yield of 80% was achieved in 48 h using 90 mg of GPC, corresponding to 164 mg of the desired product. Thus, the 1/10 molar ratio (GPC/DHA) proved to be the most suitable for achieving a high yield and good homogeneity of the substrate with the ionic liquid. This increase in yield is probably due to the fact that the high percentage of ionic liquid improves the solubilization of GPC in the reaction medium. On the other hand, it should be noted that the use of 50% MOIM-BF_4_ instead of 30% allowed us to reduce the amount of DHA required while maintaining the same yield in 48 h and optimizing its use.

### 2.6. Operational Stability of the QlowP-C18 Catalyst in the Presence of MOIM-BF_4_

The operational stability of the phospholipase biocatalyst QlowP immobilized by hydrophobic adsorption [30] on Immobeads-C18 in an ionic liquid was studied under optimal conditions (90 mg GPC and 50% MOIM-BF_4_ at 60 °C) for the synthesis of DHA-LPC. The reusability of QlowP-C18 with 30% and 50% MOIM-BF_4_ was compared to evaluate how the presence of the ionic liquid affects the stability of the enzyme.

**Figure 5:**
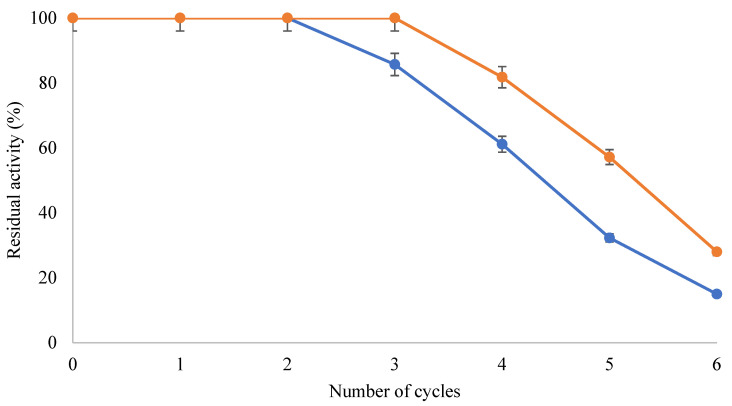
Recovered activity (%) of QlowP-C18 derivative after several cycles of reuse for the esterification reaction of GPC and DHA. Reaction conditions: 30% MOIM-BF_4_ (blue) with 1/14 substrate ratio (GPC/DHA) or 50% MOIM-BF_4_ (orange) with 1/10 (GPC/DHA), 10% enzyme derivative (*w*/*w*), and shaking at 150 rpm. In both cases, reuse was evaluated in 24-h reaction cycles at 60 °C.

Figure 5 shows the activity recovered over six consecutive 24-h reaction cycles. Cycle 0 was set as the 100% catalytic activity of QlowP-C18. For the cycles with 30% ionic liquid, the residual catalyst activity remained at 100% for two cycles, then decreased to 15% in the last cycle, with a total production of 603 mg DHA-LPC. With 50% MOIM-BF_4_, the residual activity remained at 100% for one additional cycle, reaching 28% at the end of the six cycles, with a total production of 788 mg DHA-LPC.

This experiment demonstrates that a higher percentage of ionic liquid increases the stability of the catalyst, allowing its reuse for more cycles. This suggests that MOIM-BF_4_ could stabilize the enzyme, making it an excellent alternative for the synthesis of DHA-LPC in its pure form. The optimization of the reaction with MOIM-BF_4_ has been achieved due to the advantages offered by the immobilization strategy employed for the enzyme, which results in derivatives that exhibit higher enzymatic activity compared to its free form, greater resistance to temperature increases, and higher stability in various reaction media.

### 2.7. Characterization of DHA Lysophospholipids Using NMR

With the ultimate goal of identifying which isomer of the monosubstituted DHA phospholipid was synthesized, the purification and subsequent NMR characterization of the product obtained from the esterification reaction catalyzed by QlowP-C18 in the presence of 50% MOIM-BF4 was carried out. First, the final sample was purified using solid phase extraction (SPE), obtaining eight different fractions. Fraction number 7, eluted with solvent G (chloroform: methanol [1:3]), was identified as containing the lysophospholipid DHA, with a purification yield of 60%.

**Figure 6:**
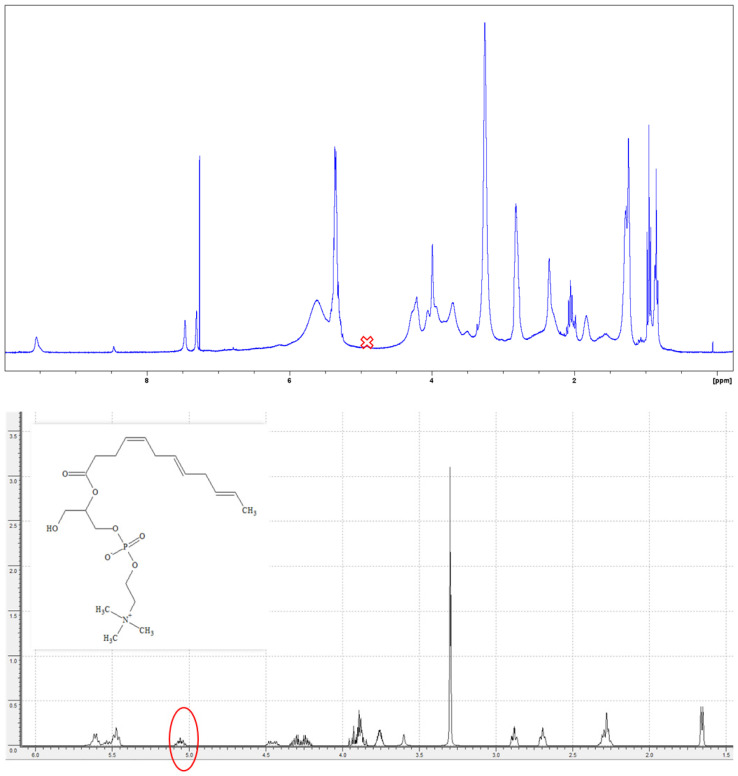
(**Top**): ^1^H-NMR spectrum obtained for the fraction containing the lysophospholipid DHA. (**Bottom**): Predicted ^1^H-NMR spectrum for the lysophospholipid with DHA at the sn-2 position.

Subsequently, the proton nuclear magnetic resonance (^1^H-NMR) spectrum of fraction 7 was analyzed. In particular, carbon 2 provides key information to distinguish between isomers. Figure 6 presents two spectra: the first shows the experimental ^1^H-NMR spectrum, while the second is the predicted spectrum for the lysophospholipid with DHA esterified at the sn-2 position, according to the ACD/Labs program.

**Figure 7:**
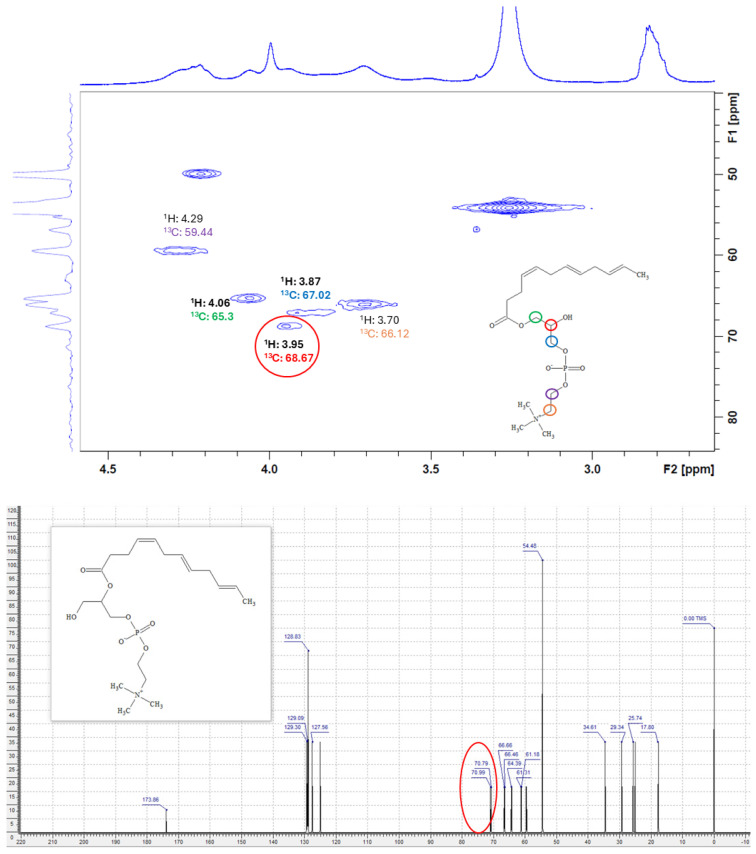
(**Top**): Two-dimensional ^1^H-^13^C HSQC spectrum (*y*-axis: ^13^C vs. *x*-axis: ^1^H) obtained for the fraction containing the lysophospholipid DHA. (**Bottom**): Predicted ^13^C-NMR spectrum for the lysophospholipid with DHA at the sn-2 position.

Comparative analysis reveals that the expected signal around 5.05 ppm, which would indicate DHA at the sn-2 position, was not observed. This suggests that DHA is esterified at the sn-1 position. Given the complexity of the molecule and the high similarity of its positional isomers, the theory was verified by two-dimensional HSQC ^1^H-^13^C analysis of the same sample. Figure 7 shows the results obtained from the ^13^C versus ^1^H analysis for fraction 7, which contains the lysophospholipid DHA.

In the two-dimensional ^1^H-^13^C HSQC spectrum of the analyzed compound, all the ^13^C and ^1^H signals correspond to the structure of the lysophospholipid with DHA at the sn-1 position. Specifically, a ^13^C signal at 68.67 ppm and a ^1^H signal at 3.95 ppm are identified, which are characteristic of the acyl-free position at carbon 2 of the phospholipid, featuring a single hydroxyl group (-OH). Compared to the ^13^C spectrum predicted by the ACD/Labs program, where a clear peak is expected around 71 ppm when the fatty acid is esterified at position 2 of the phospholipid, this signal does not appear in the experimental ^1^H-^13^C HSQC spectrum obtained. In addition, the signal associated with the proton at the sn-2 position at 5.05 ppm was not detected.

Therefore, the spectroscopic data confirm that the lysophospholipid synthesized is sn1-DHA-LPC, i.e., the isomer with DHA at the sn-1 position.

## 3. Materials and Methods

### 3.1. Materials

Docosahexaenoic acid (DHA) was obtained from the enzymatic hydrolysis of anchovy oil, purchased in capsules from NuaBiological in a pharmacy in Madrid (Spain). Glycerophosphocholine (GPC) was purchased from Cayman Chemical Company (Ann Arbor, MI, USA). Soluble lipases such as Lipozyme^®^ TL from *Thermomyces lanuginosa* (TLL), *Candida antarctica B* (CALB), Palatasa^®^ 20,000 L (PALA), Eversa^®^ Transform 2.0 (NS40), Novozym^®^40119 (NOVO) and phospholipases Lecitase^®^ Ultra (LECI), and Quara^®^ LowP (QlowP) were kindly donated by Novozymes (Bagsvaerd, Denmark). Immobeads-C18 IB-ADS-3 (C18) immobilization support was provided by ChiralVision (Leiden, the Netherlands), and DEAE-Sepharose purification support was from Sigma–Aldrich. ExtraBond^®^ Silica columns were obtained from Scharlab, S.L. (Barcelona, Spain). Hexadecyltrimethylammonium bromide (CTAB), sodium chloride, p-nitrophenyl butyrate (pNPB), and 3Å pore size molecular sieve (2–3 mm bead) were provided by Sigma–Aldrich. Methanol, chloroform, hexane, 2-propanol, ethyl acetate, ammonium hydroxide, and acetonitrile were purchased from VWR Chemicals (Matsonford Road Radnor, PA, USA) and Sigma–Aldrich. 1,2-didocosahexaenoyl-sn-glycero-3-phosphocholine (Di-DHA-PC) standard was purchased from Avanti Polar Lipids (Birmingham, AL, USA). The ionic liquids (ILs) used were 1-butyl-3-methylimidazolium tetrafluoroborate (BMIM-BF_4_), 1-ethyl-3-methylimidazolium acetate (EMIM-Ac), 1-ethyl-3-methylimidazolium chloride (EMIM-Cl), 1-ethyl-3-methylimidazolium hexafluorophosphate (EMIM-PF_6_), 1-ethyl-3-methylimidazolium tetrachloroaluminate (EMIM-AlCl_4_), 1-ethyl-3-methylimidazolium tetrafluoroborate (EMIM-BF_4_), 1-ethyl-3-methylimidazolium trifluoromethanesulfonate (EMIM-Otf), and 1-methyl-3-octylimidazolium tetrafluoroborate (MOIM-BF_4_), all provided by Sigma–Aldrich. All other reagents and solvents used were of analytical grade.

### 3.2. Methods

#### 3.2.1. Determination of the Activity of Different Soluble and Immobilized Lipases

The activity assay was carried out using a spectrophotometer with a thermostatted cell and continuous magnetic stirring (500 rpm) for 2 min. The increase in absorbance at 348 nm (ε = 5150 M^−1^cm^−1^) produced by p-nitrophenol (pNP) released after the hydrolysis of the 0.4 mM p-nitrophenyl butyrate (pNPB) substrate in 25 mM sodium bicarbonate at pH 8.5 was measured at 25 °C. In the case of phospholipase QlowP, this increase was measured in 10 mM sodium phosphate buffer at pH 7. To initiate the reaction, 0.1 mL of soluble lipases/phospholipases (blank or supernatant) and their immobilized preparations (suspension) were added to 2.5 mL of the substrate solution. Enzyme activity was calculated as µmol of pNPB hydrolyzed per minute per mg of enzyme (IU) under the described conditions.

#### 3.2.2. Immobilization of Lipases/Phospholipases on the Hydrophobic Support Immobeads-C18

Five different lipases (TLL, NS40, PALA, CALB, and NOVO) and two phospholipases (LECI and QlowP) were immobilized on the hydrophobic support Immobeads-C18 by interfacial adsorption. These enzymes adsorb on this type of support in their open and hyperactive form [36]. Using the Immobeads-C18 support, enzyme preparations with maximum loading were obtained. To achieve this maximum loading, the protocol established in a previous study [30] was followed. However, the NOVO-C18 and QlowP-C18 derivatives were not previously studied; their optimization was achieved in this study.

On the one hand, NOVO purification was carried out using DEAE-Sepharose support by ion exchange. For this purpose, 20 g of DEAE support was added to a buffered solution (10 mM sodium phosphate buffer at pH 7) containing 1000 mg of enzyme (38 mg/mL according to the results of the Bradford assay). Purification was carried out for 24 h at 25 °C with continuous shaking. The activity of the suspension and supernatant was measured periodically using the p-NPB assay to monitor the process. Once all the enzyme was adsorbed on the DEAE, desorption was performed using a 0.1 M NaCl solution in 148 mL of phosphate buffer. The DEAE support containing the purified enzyme was added to this solution and stirred for 2.5 h, achieving total desorption of the enzyme.

The supernatant containing the purified enzyme was then collected for immobilization. To this volume of purified enzyme, 0.01% CTAB was added. Subsequently, 5 g of pre-moistened Immobeads-C18 immobilization support was introduced. The mixture was kept at 25 °C with continuous stirring for 48 h. The p-NPB assay was performed to monitor the immobilization process.

Moreover, QlowP-C18 was also purified using DEAE-Sepharose support by ion exchange. For this purpose, 20 g of DEAE support was added to a buffer solution containing 1000 mg of enzyme in 10 mM sodium phosphate buffer at pH 7 (equivalent to 6 mg/mL according to the results of the Bradford assay). Purification was carried out for 24 h at 25 °C with continuous shaking. The activity of both the suspension and the supernatant was measured periodically using the p-NPB assay to monitor progress. Once all the enzyme was adsorbed on the DEAE support, desorption was initiated using a 0.1 M NaCl solution in 158 mL of phosphate buffer. The DEAE support containing the purified enzyme was introduced and shaken for 2.5 h, resulting in complete desorption of the enzyme.

The supernatant containing the purified enzyme was then collected for immobilization. To this volume of purified enzyme, 0.02% CTAB was added. Subsequently, 5 g of pre-moistened Immobeads-C18 immobilization support was introduced. The mixture was kept at 25 °C with continuous stirring for 96 h. The p-NPB assay was performed throughout the immobilization process to monitor the activity of the enzyme.

#### 3.2.3. Drying of Lipases/Phospholipases Derivatives

The enzyme derivative was washed and dried before use since it was used in reactions in an anhydrous medium. Accordingly, the derivative was washed with 50 mL of a mixture of water and acetone, with increasing percentages of acetone, using a sintered glass funnel until completely dry. For each gram of derivative, 50 mL of each of the following solutions were used: 100% water; water–acetone mixtures (70:30, 50:50, and 30:70, *v*/*v*); and 100% acetone.

#### 3.2.4. Enzymatic Synthesis of Structured Phospholipids from Docosahexaenoic Acid

The esterification reaction with docosahexaenoic acid was carried out in a medium containing ionic liquids. The initial reaction mixture consisted of 250 mg of dried lipase/phospholipase derivative, 30 mg of GPC, 0.25 g of molecular sieve with a pore size of 3 Å, 1.75 mL of free DHA, and 0.75 mL of ionic liquid. Free DHA was obtained through the enzymatic hydrolysis of anchovy oil, following the method described in [19].

To optimize reaction conditions, we investigated the addition of various percentages of ionic liquids relative to the final volume of 2.5 mL, along with increasing amounts of GPC. The reactions were carried out in 5 mL glass vials at 60 °C with constant stirring in an incubator at 150 rpm. Prior to the addition of the derivative, the vials were incubated and shaken for 15 min to increase the solubility of GPC in the reaction medium. For each experiment, a negative control without lipase/phospholipase derivative was performed under the same conditions. Phospholipid synthesis of DHA was monitored using TLC (using the same mobile phase described at [30]) and then quantified using HPLC by sampling the supernatant. For TLC, 60 μL of the reaction medium was taken and diluted in 900 μL of chloroform, and then, for HPLC, 100 μL of this solution was taken and diluted in 900 μL of methanol/acetonitrile (80:20 *v*/*v*; the mobile phase used in HPLC). Experiments were performed in triplicate, and the standard deviation was always less than 5%.

#### 3.2.5. Recycling Cycles of Lipases/Phospholipases Derivatives

For recycling studies, the enzymatic esterification reaction was carried out in glass vials (final volume 2.5 mL) at 60 °C in an anhydrous medium containing the ionic liquid identified as the most suitable. The process consisted of reusing the derivative for as many cycles as possible under the most favorable reaction conditions. After reaching the optimum reaction time, the reaction mixture was carefully filtered to recover the catalyst for reuse. To effectively separate the derivative from the reaction medium, it was washed and dried several times with hexane and acetone to remove any remaining substrates or products. Subsequently, new substrates (DHA and GPC) and ionic liquid were added along with the reused catalyst. The weight of the derivative was carefully controlled to avoid losses in successive cycles, and the reaction was repeated under the same conditions. This process was repeated as many times as possible until the enzyme completely lost its activity. The yield of DHA lysophospholipids (DHA-LPC) obtained in the first reaction was set at 100%, and the yield in subsequent reactions was calculated accordingly. Analysis of the product peaks was performed using HPLC.

#### 3.2.6. Quantitative Analysis Using High-Performance Liquid Chromatography (HPLC)

**Figure 8:**
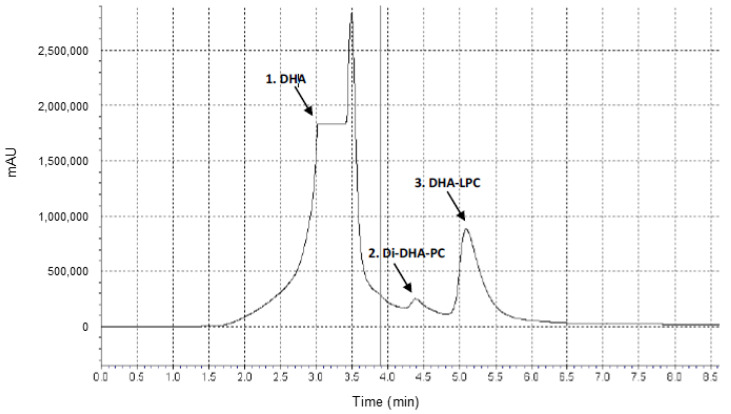
Chromatogram of the synthesis profile of the disubstituted phospholipid Di-DHA-PC and the monosubstituted DHA-LPC at 48 h of reaction. The peaks identified were: 1. DHA substrate, 2. Di-DHA-PC, 3. DHA-LPC.

High-performance liquid chromatography analyses were performed with an NP-HPLC system (Spectra Physic SP 100 pump coupled to a Spectra Physic SP 8450 UV detector) employing a normal phase column (LiquidPurple Sil, 250 × 4.6 mm, 5 μm) with a prefilter column (0.5 μm disk and gasket). In addition, aliquots were prefiltered using a 4 mm/0.45 μm PVDF syringe filter. Elution of the products occurred at a flow rate of 1 mL/min using a methanol/acetonitrile mobile phase (80:20 *v*:*v*). UV detection was set at 205 nm due to the double bond content of the molecules, which promotes significant absorbance at that wavelength. Synthesis yields were calculated from the peak areas corresponding to different concentrations of phosphatidylcholine DHA and lysophosphatidylcholine, with retention times of 4.4 min (Di-DHA-PC) and 5.1 min (DHA-LPC), respectively (Figure 8). HPLC analyses were performed in triplicate, and the standard deviation was consistently kept below 5%.

#### 3.2.7. Purification of Phospholipids by Solid-Phase Extraction (SPE)

The reaction products were isolated from the reaction mixture using solid phase extraction (SPE) with a polar stationary phase. ExtraBond silica columns^®^ were preconditioned with 2 mL of hexane. The sample (50 µL reaction medium/150 µL hexane) was allowed to adsorb onto the matrix by gravity percolation through the cartridge. The SPE column was eluted with 2 mL of solvent A (hexane), 2 mL of solvent B (hexane: ethyl acetate [9:1]), 2 mL of solvent C (hexane: ethyl acetate [7:3]), 4 mL of solvent D (hexane: isopropanol [5:5]), 4 mL of solvent E (hexane: isopropanol [3:7]), 4 mL of solvent F (hexane: isopropanol [1:9]), 4 mL of solvent G (chloroform: methanol [1:3]), and 4 mL of solvent H (chloroform: methanol [1:6]). The compounds were eluted from lowest to highest polarity, which required eight fractionation steps to remove all the reaction mediums used and to obtain a pure DHA-LPC fraction. The fractions eluted from the SPE column were dried under nitrogen and redissolved for TLC and HPLC analysis. The protocol described was adapted from the literature references [37,38].

#### 3.2.8. Nuclear Magnetic Resonance (NMR) Characterization

The final product (10 mg) obtained after the SPE technique was mixed with 0.2 mL CDCl3 and transferred to a 5 mm NMR tube. Subsequently, ^1^H-NMR was used to detect the lysophospholipid composition of DHA in the purified reaction product. A fully digital nuclear magnetic resonance spectrometer (Avance III-HD NANOBAY 300 MHz, Bruker, Karlsruhe, Germany) with deuterated solvent and referenced to tetramethylsilane (TMS) was used. Test conditions included a probe temperature of 25 °C, a spin and pulse of 9 µs, 32 scans, and a relaxation delay (d1) of 1 s.

Next, using the same sample, the directly bonded ^1^H and ^13^C nuclei were correlated in the ^1^H-^13^C Heteronuclear Simple Quantum Coherence (HSQC) spectrum. For the analysis of the core of ^13^C, a 90° pulse of 10 µs was used. Signal shifts were indicated in ppm, and the integrated relative intensity of each peak was used to calculate the composition of the product mixture. Both one-dimensional ^1^H-NMR spectroscopy and two-dimensional ^1^H-^13^C HSQC spectroscopy were carried out at the Nuclear Magnetic Resonance Laboratory of the Interdepartmental Research Service (SIdI) of the Autonomous University of Madrid (UAM). Molecular structure prediction and elucidation from the NMR spectra obtained were performed using ACD/Labs computational software at the Bioengineering Institute (IB) of the Miguel Hernández University of Elche (UMH).

## 4. Conclusions

The presence of ionic liquid in the reaction medium maintains the sn-1,3 regioselectivity of the different enzymes studied, producing exclusively the monosubstituted phospholipid DHA. Among the biocatalysts evaluated, QlowP-C18 was the most promising when used with 30% MOIM-BF_4_, achieving an 80% yield of DHA-LPC in 48 h. Several ionic liquids with different physicochemical properties were investigated to determine their efficacy in the esterification of GPC and DHA. It was observed that MOIM-BF_4_ offered the best performance, probably because it improves the solubility of GPC in DHA. This hydrophilic solvent significantly increased the solubility of GPC, allowing the same maximum yield of 80% in 48 h but with an elevated MOIM-BF_4_ concentration of 50%.

The use of 50% MOIM-BF4 optimizes the compound formation rate to 20.06 (mg/mL.h) × g and minimizes the excess of DHA in the medium. In addition, this percentage also improves the stability of the QlowP-C18 biocatalyst, maintaining 100% of its residual activity for an additional cycle (totaling three cycles) compared to a lower percentage of ionic liquid (30% MOIM-BF_4_). At the end of six cycles, a total production of 788 mg of DHA lysophospholipid was achieved. Therefore, it can be concluded that the use of 50% MOIM-BF_4_ as a solvent in the reaction medium provides increased stability to the phospholipase derivative QlowP-C18.

These results have been achieved by using the highly active and stable immobilized phospholipase biocatalyst of maximum enzyme loading, capable of operating under adverse reaction conditions such as highly hydrophobic anhydrous systems, highly viscous solvent media, and high reaction temperatures. NMR analysis confirmed that the DHA lysophospholipid synthesized is the sn1-DHA-LPC isomer. Although DHA at the sn-2 position is preferred to increase the DHA content in the brain [39,40], recent studies suggest that DHA at the sn-1 position may also contribute to memory enhancement [41].

## Figures and Tables

**Table 1:** Molar ratio of substrates (GPC/DHA) and initial reaction rate, V1 (mg/mL of DHA-LPC per hour and g of derivative with 10% conversion) for QlowP-C18-catalyzed synthesis of mono-substituted phospholipids DHA with 90 mg of GPC at 60 °C for different percentages of MOIM-BF_4_.

MOIM-BF_4_: DHA (%)	GPC/DHA (mol)	V1 (mg/mL·h) × g
0%: 100%	1/21	16.35
10%: 90%	1/18	17.43
30%: 70%	1/14	18.61
50%: 50%	1/10	20.06
70%: 30%	1/6	11.76
90%: 10%	1/2	3.11

## Data Availability

The authors are unable or have chosen not to specify which data have been used.
